# Supplementary material for: Bandgap Engineering via Doping Strategies for Narrowing the Bandgap below 1.2 eV in Sn/Pb Binary Perovskites: Unveiling the Role of Bi3+ Incorporation on Different A-Site Compositions
Source: Nanomaterials (Basel). 2024 Sep 26;14(19):1554. doi: 10.3390/nano14191554 (PMC11478006; doi:10.3390/nano14191554)
Supplement: Supplementary file 1 [file nanomaterials-14-01554-s001.zip › nanomaterials-3210387-supplementary.pdf]

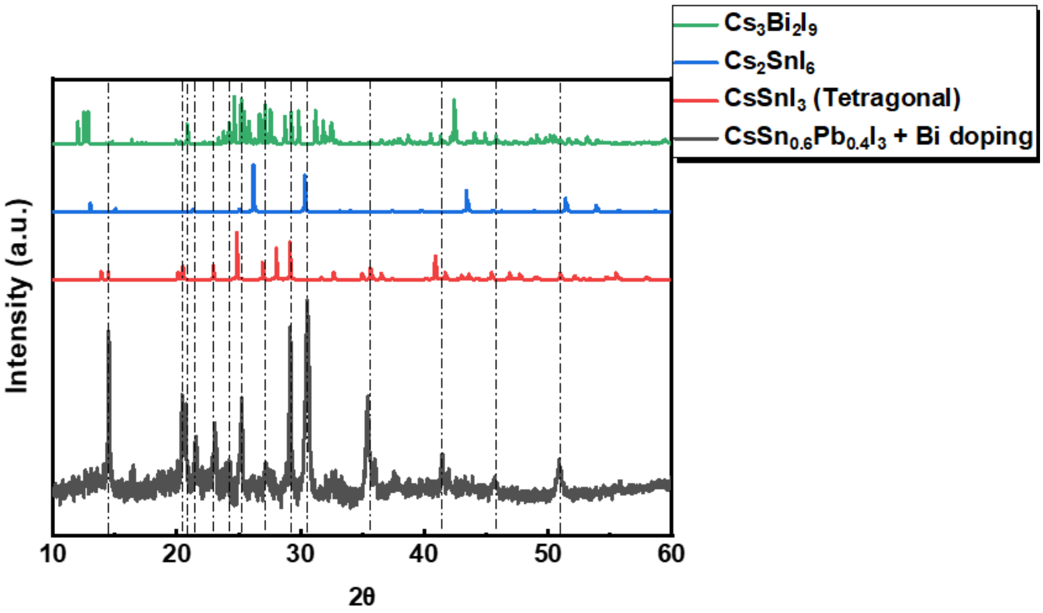

Figure S1. XRD patterns of Bi-doped CSPI perovskites along with simulated patterns for $CsSnI_3$, Cs_2SnI_6, and $Cs_3Bi_2I_9$.

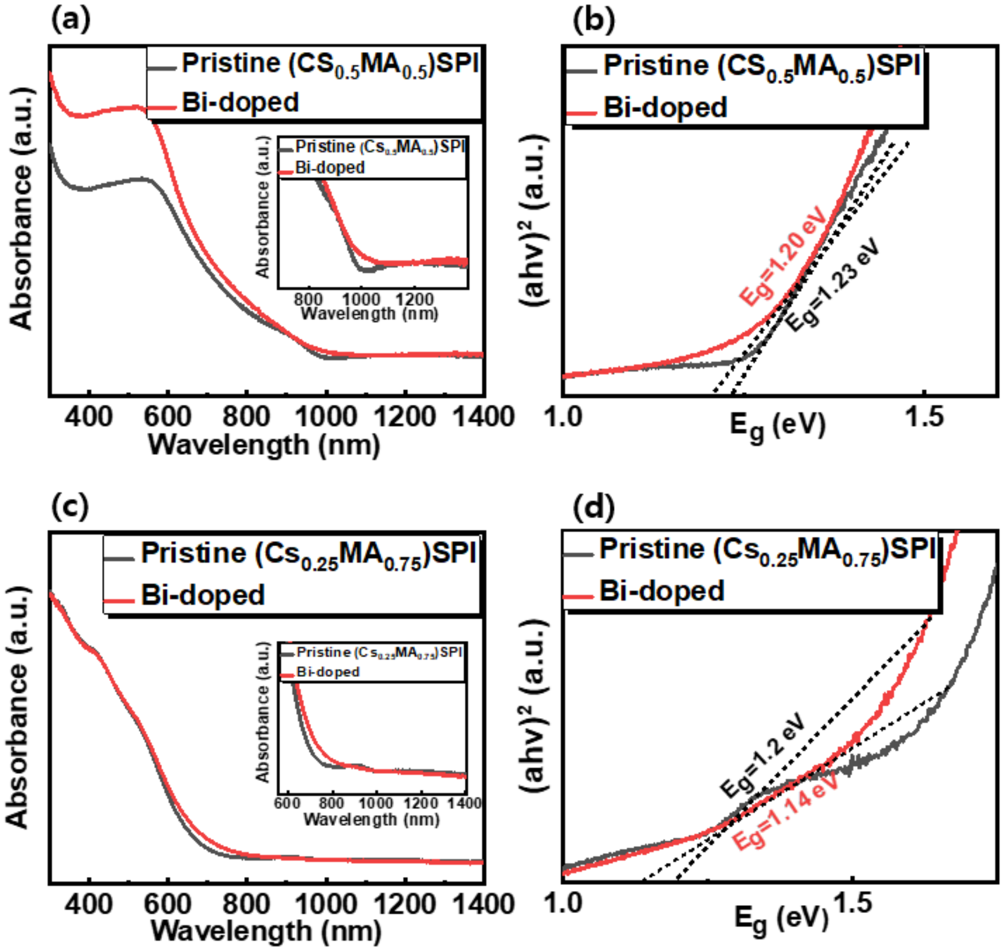

Figure S2. (a) Absorption spectra of pristine and Bi-doped $Cs_{0.5}MA_{0.5}SPI$ perovskite films, with the inset highlighting the enlarged absorption spectrum, (b) Tauc plot of pristine and Bi-doped $Cs_{0.5}MA_{0.5}SPI$ perovskite films, (c) Absorption spectra of pristine and Bi-doped $Cs_{0.25}MA_{0.75}SPI$

perovskite films, with the inset highlighting the enlarged absorption spectrum, (d) Tauc plot of pristine and Bi-doped $Cs_{0.25}MA_{0.75}SPI$ perovskite films.

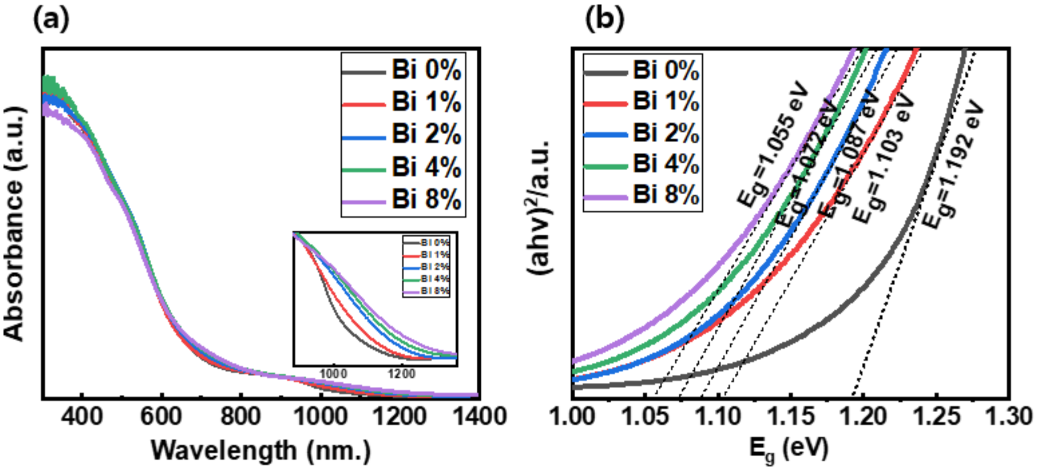

Figure S3. (a) Absorption spectra of pristine and Bi-doped (1%, 2%, 4%, 8%) MSPI perovskite films, (b) Tauc plots for pristine and Bi-doped (1%, 2%, 4%, 8%) MSPI perovskite films.

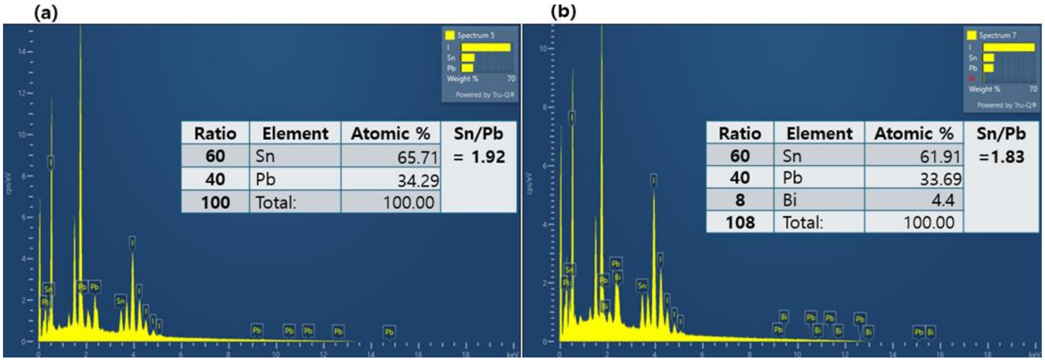

Figure S4. (a) EDS results of pristine MSPI and (b) Bi-doped MSPI perovskite films.

Table S1. Bismuth concentration and tolerance factor of pristine and Bi-doped CSPI and MSPI perovskite films.

	Bismuth concentration [%]	Tolerance Factor [T]
CSPI	0	0.8525
	1	0.8499
MSPI	0	0.9131
	1	0.9103

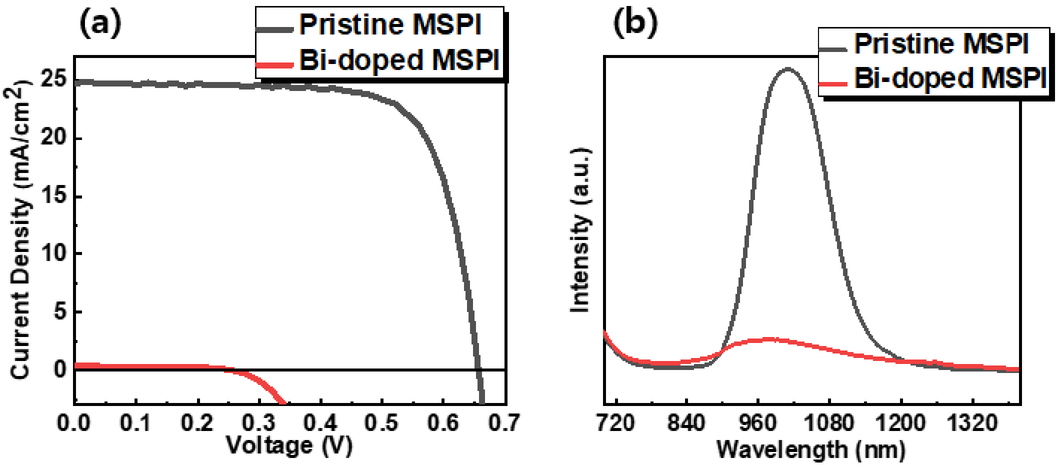

Figure S5. (a) J-V curves of pristine and Bi-doped MSPI perovskite devices, (b) Steady-state PL spectra of pristine and Bi-doped MSPI perovskite devices.

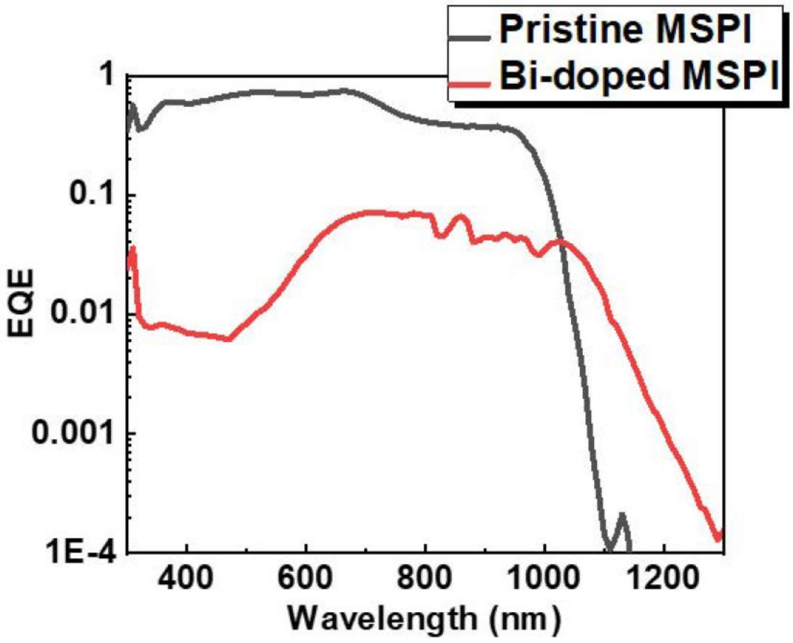

Figure S6. EQE spectra of pristine and Bi-doped MSPI devices.
